# The Mental Burden of the COVID-19 Pandemic: A Retrospective Post-pandemic View From a Romanian Sample

**DOI:** 10.7759/cureus.72631

**Published:** 2024-10-29

**Authors:** Adriana Mitrea, Anca Hăisan, Ani Cășărică, Rodica Gabriela Enache, Elena Danteș

**Affiliations:** 1 Pulmonology Department, Constanta County Clinical Emergency Hospital ‘St. Apostol Andrei’, Faculty of Medicine, ‘Ovidius’ University, Constanța, ROU; 2 Emergency Medicine Department, Faculty of Medicine, ‘Grigore T Popa’ University of Medicine and Pharmacy, Iași, ROU; 3 Emergency Department, “Sf. Spiridon” County Clinical Emergency Hospital, Iași, ROU; 4 Psychology Department, County Center for Resources and Educational Assistance, Constanța, ROU; 5 Psychology Department, Psycho-pedagogical Assistance Office, "Remus Opreanu" Secondary School, Constanța, ROU; 6 Faculty of Psychology, ‘Ovidius’ University, Constanța, ROU; 7 Faculty of Medicine, ‘Ovidius’ University, Constanta, ROU; 8 Pulmonology Department, Clinical Hospital of Pneumophtisiology, Constanța, ROU

**Keywords:** anxiety, covid-19, covid-19 reinfection, cross-sectional, dass-21, depression, mental health, psychological distress, romanian population, stress

## Abstract

Background: The enduring psychological effects of the COVID-19 pandemic continue to affect individuals long after the immediate health concerns have subsided. This research aims to identify specific groups within the Romanian population who are at a higher risk of experiencing mental health challenges that can interfere with everyday life and may lead to more serious mental health disorders.

Methods: Conducted as a cross-sectional survey, this study assessed the severity of psychological distress using the Depression, Anxiety, and Stress Scale-21 (DASS-21) questionnaire in relation to COVID-19-related information and socio-demographic data to investigate the factors associated with psychological distress during the COVID-19 pandemic in Romania.

Results: Analysis of 521 questionnaires, filled out by a predominantly middle-aged cohort of 320 women and 201 men (mean age = 42.24, SD = 11.96), revealed that 63.72% of participants worked outside the healthcare field. Those unemployed or retired reported significantly higher anxiety levels than individuals in other sectors. Moreover, living alone, experiencing the loss of close relatives (6.14%) or friends (33.59%), and undergoing hospitalization or reinfection due to COVID-19 were linked to significantly elevated distress scores.

Conclusion: By identifying the segments of the population most vulnerable to psychological distress, as evidenced by higher scores among the unemployed, retirees, individuals living alone, and those directly affected by COVID-19 through personal health or loss, targeted initiatives for psychological screening and therapy can be established. Such measures are essential for enhancing the post-pandemic mental well-being of Romanians, addressing the specific needs uncovered in this study. These findings are limited by the study type and sample size; therefore, more extensive, longitudinal research conducted on a larger population sample is necessary.

## Introduction

Since the beginning of the pandemic, medical professionals, psychologists, and health researchers have highlighted the risks of psychological distress among the general population and healthcare workers exposed to COVID-19 pathology [1-4]. Even in the absence of illness, population quarantine has had adverse psychological effects, as evidenced in previous epidemics (MERS (Middle East respiratory syndrome) and SARS (severe acute respiratory syndrome)) and in the recent pandemic. These effects are particularly pronounced during prolonged isolation, inadequate communication, and instances of supply shortages and financial strain [5,6]. Long-term studies on the psychiatric aftermath of the SARS epidemic revealed psychological vulnerabilities that persisted for up to four years after the initial outbreak [7]. When examining the repercussions of reinfections, it has been found that the heightened risks extend to all adverse health outcomes, including mental health issues, during both the acute and post-acute phases of reinfection [8,9]. 

While previous research has explored the psychological impact of COVID-19 infection and quarantine measures, limited attention has been given to the long-term psychological consequences of reinfection, particularly within specific cultural and societal contexts such as Romania. In Romania, there is a strong scientific interest in studying the impact of COVID-19 from both medical [10] and psychological perspectives [11-14]. However, the psychological consequences of reinfection with this new pathology and the timing of reinfection concerning therapeutic possibilities have not yet been addressed within this sociocultural context.

Given the significant and continuously evolving impact of the pandemic on mental health, with effects persisting well beyond the initial outbreak [15], our study seeks to explore psychological distress among a sample of Romanian citizens. Tracking the psychological impact of the pandemic, even at a temporal distance, makes sense in terms of identifying the imprint of distressed memory. Thus, we can identify the profile of psychological fragility and, subsequently, act through specific proactive interventions.

The research aims to examine the following scientific hypotheses:

1. The psychological burden during the COVID-19 pandemic varied according to occupation and the extent of social involvement. Specifically, individuals who were actively employed or retired experienced different levels of stress, with healthcare workers facing particularly high psychological pressure. 2. Romanians living with their families experienced a reduced psychological impact compared to those living alone. Moreover, we propose that having a pet serves as a protective factor, offering emotional support. 3. Anxiety, stress, and depression induced by the pandemic were more severe in individuals who experienced the death of a friend or relative. 4. Vaccination provided an emotionally protective effect by reducing stress, anxiety, and depression levels. 5. The hospitalization of individuals with COVID-19 led to increased psychological distress, and the persistence of symptoms further deteriorated the mental health of Romanians. 6. Experiencing the illness was associated with a significant negative emotional impact, which varied across the three phases of the pandemic. 7. Reinfection with SARS-CoV-2 placed an additional emotional strain on the population we analyzed. Thus, the purpose of the study is to uncover the complex relationships between the various determinants.

A draft of this article was previously posted to the Preprint.org server [16].

## Materials and methods

Study design

An online survey was sent to adult patients in southeastern Romania through their general practitioners' and pulmonologists' electronic health records. The questionnaire was open for responses for 30 days. Exclusion criteria for the study included individuals under 18 years of age (in Romania, individuals above 18 years are considered adults) and those with declared psychiatric pathology. It is important to note that respondents did not experience any benefits or secondary repercussions as a result of participating in this study. Their participation was voluntary and without constraints, and non-completion of the interview resulted in exclusion from statistical processing.

The study adhered to the relevant legislation governing the processing and free movement of personal data. This research aligns with international ethical recommendations, ensuring absolute confidentiality of the collected data and upholding the anonymity and security of participants. The institutional ethics committee at "Ovidius" University of Constanța, Romania, with protocol number DCI 54/31.05.2023, granted approval for this study. The principles outlined in the Declaration of Helsinki were appropriately adhered to. Following informed consent, participants completed a 10-minute online survey.

Measures

The questionnaire underlying our study encompassed socio-demographic data (age, gender, social status (employed/retired, living arrangements, pet ownership), questions related to COVID-19 pathology (experience of the illness, reinfection status, vaccination history, hospitalization, persistent symptoms, loss of a relative or friend during the pandemic, timing of illness about the three pandemic waves in Romania), and the Depression, Anxiety, and Stress Scale-21 (DASS-21) questionnaire. 

The DASS-21 serves as a widely utilized assessment tool in both general and clinical populations globally [17]. The study utilized the short version of the self-report scale, consisting of 21 questions. Each question was rated on a 4-point Likert scale, ranging from 0 (does not apply to me at all - NEVER) to 3 (applies to me very often or most of the time - ALWAYS). The total score was computed by summing up the points for each disease category, with each category comprising seven questions. Measuring negative emotional states is crucial for clinicians to recognize and address mental disorders. Elevated levels indicative of negative affect act as a warning sign, underscoring the necessity for proactive intervention measures to prevent the development of psychiatric disorders. Based on their responses, emotional distress could be categorized as normal, mild, moderate, severe, or extremely severe. A higher score indicates a more severe manifestation of the respective disorder. We must mention that the DASS-21 questionnaire has been validated as a psychological analysis tool for the population in Romania [17,18].

Statistical analysis

The study data was analyzed utilizing IBM SPSS Statistics 25 software (IBM Corp., Armonk, NY), and data visualizations were generated using Microsoft Office Excel/Word 2021. For quantitative variables, normal distribution was assessed through the Shapiro-Wilk test, and outcomes were presented as means with standard deviations or medians with interquartile ranges. Qualitative variables were expressed as counts or percentages, and group distinctions were evaluated using Fisher's exact test. To provide additional insights into the contingency table results, Z-tests with Bonferroni correction were conducted. Quantitative independent variables lacking a normal distribution were compared between groups using either the Mann-Whitney U test or the Kruskal-Wallis H test. Post-hoc Dunn-Bonferroni tests were performed for more in-depth analysis and interpretation of the results in the comparisons of quantitative independent variables. Generalized univariable and multivariable linear models were used to examine the relationship between the analyzed scores and possible risk factors, and the performance of the prediction for each factor was calculated using the beta coefficient with 95% confidence intervals.

## Results

After collecting 534 responses, 521 were deemed valid for analysis. The remaining responses were excluded due to objective reasons.

Among the respondents, there were 201 males and 320 females, spanning ages 18 to 85 years. To better characterize the cohort, we categorized them as young adults (ages 18-24), adults (ages 25-35), middle-aged individuals (ages 36-64), and older adults (ages >65). Females constituted 61.42% of the participants, while males accounted for 38.58%. A majority of the participants fell into the middle-aged category (n = 296; 56.81%), with substantial participation from adults (n = 168; 32.24%). Young adults (n = 37; 7.10%) and older adults (n = 20; 3.83%) comprised smaller proportions of the sample. The mean age in the study group was 42.24 ± 11.96 years.

The survey used questions from the DASS-21 questionnaire, which is designed to assess the presence of symptoms of anxiety, stress, and depression in a generally healthy population (Table 1). Each condition is assessed using seven questions resulting in scores between 0 and 21. As the shortened version of the questionnaire was used in this survey, the scores obtained will be doubled and subsequent analysis will determine the severity of these symptoms.

**Table 1:** Illustration of the DASS according to severity DASS, Depression Anxiety Stress Scale. Ref: [17].

Severity	Depression	Anxiety	Stress
Normal	0-9	0-7	0-14
Mild	10-13	8-9	15-18
Moderate	14-20	10-14	19-25
Severe	21-27	15-19	26-33
Extremely severe	28+	20+	34+

For the translated and validated version for the population in Romania, the DASS-21 demonstrated strong internal consistency. Anxiety Cronbach’s α was 0.80; depression was 0.80; stress was 0.77; overall was 0.88 [18].

Comparative analysis of anxiety, depression, and stress levels based on occupational status

**Figure 1:**
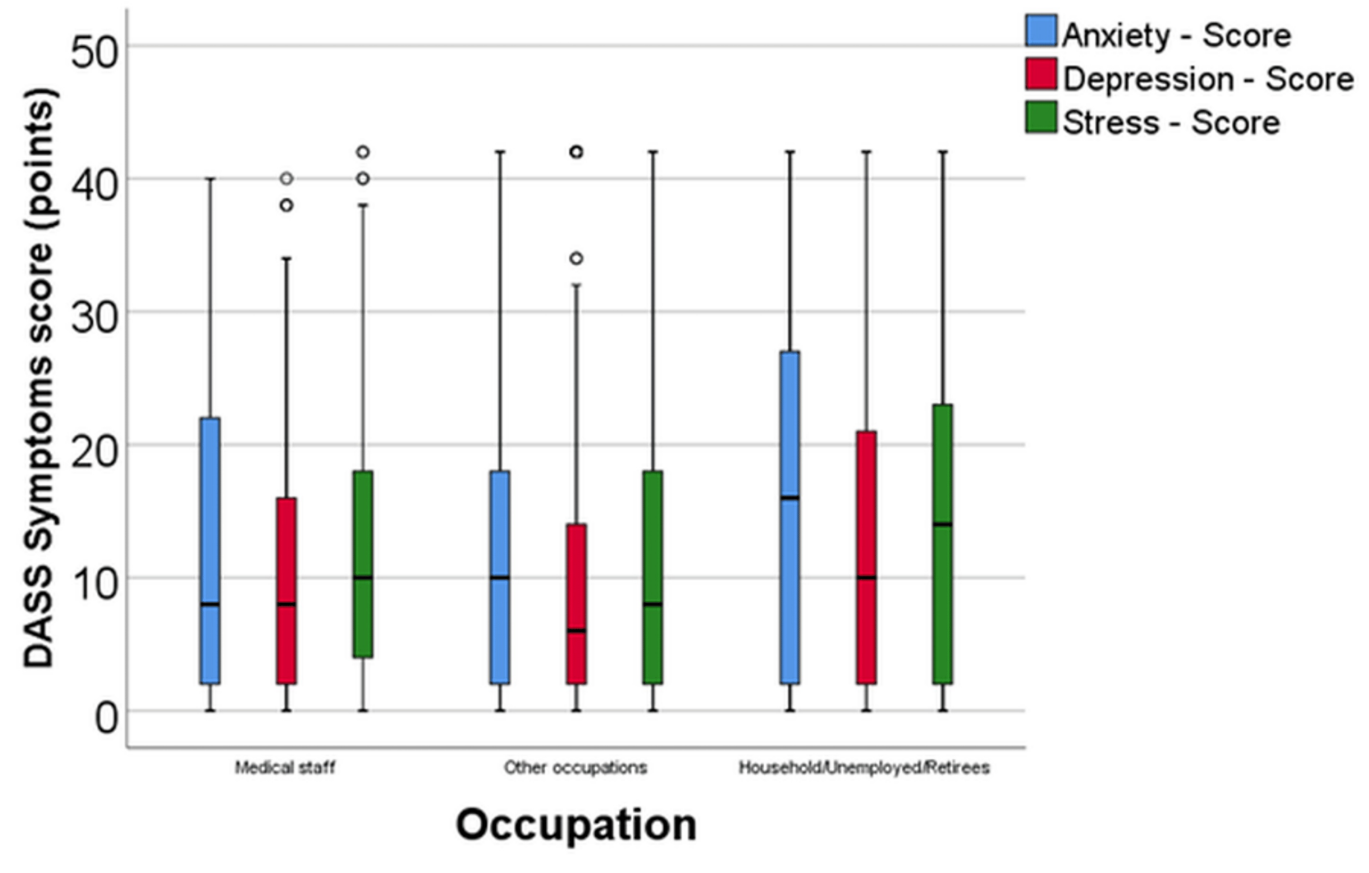
. Comparison of anxiety, depression, and stress scores across different occupational activities DASS, Depression, Anxiety, and Stress Scale.

We analyzed, in terms of DASS parameters, the emotional impact of the pandemic distributed based on the type of occupational activity. We paid special attention to individuals working in the medical field, who not only experienced the pressure of working in an infectious risk environment but also witnessed the suffering of COVID-19 patients (Figure 1).

According to the Shapiro-Wilk test, the distribution of values was non-parametric across all groups (p < 0.05). Most participants were employed outside the medical field (n = 332; 63.72%), while those in the medical field constituted 24.18% (n = 126), and the unemployed or retired accounted for 12.09% (n = 63). Based on Kruskal-Wallis H-tests, there were significant differences in anxiety (p = 0.040), depression (p = 0.049), and stress (p = 0.027) scores among the groups. However, post-hoc Dunn-Bonferroni tests revealed significant differences only in anxiety scores: Individuals working as domestic workers, being unemployed, or retired exhibited a significantly higher anxiety score (median = 16, IQR = 2-28) compared to those employed in various other occupational domains (median = 10, IQR = 2-18) (p = 0.035). A similar statistical pattern was observed for depression or stress scores, although, in these cases, only trends toward statistical significance were noted (depression - p = 0.079, stress - p = 0.061), potentially due to the limited number of household members included in the analysis.

Comparative analysis of anxiety, depression, and stress levels based on living arrangements

Humans are social beings who communicate affectionately, empathize, and show compassion toward their fellow beings. Based on these premises, we interviewed respondents regarding whether they live alone or with other family members. We applied DASS variables over this pragmatic aspect, which holds significant influence over mental well-being, especially in crises, during travel restrictions, and interpersonal socialization limitations.

**Figure 2:**
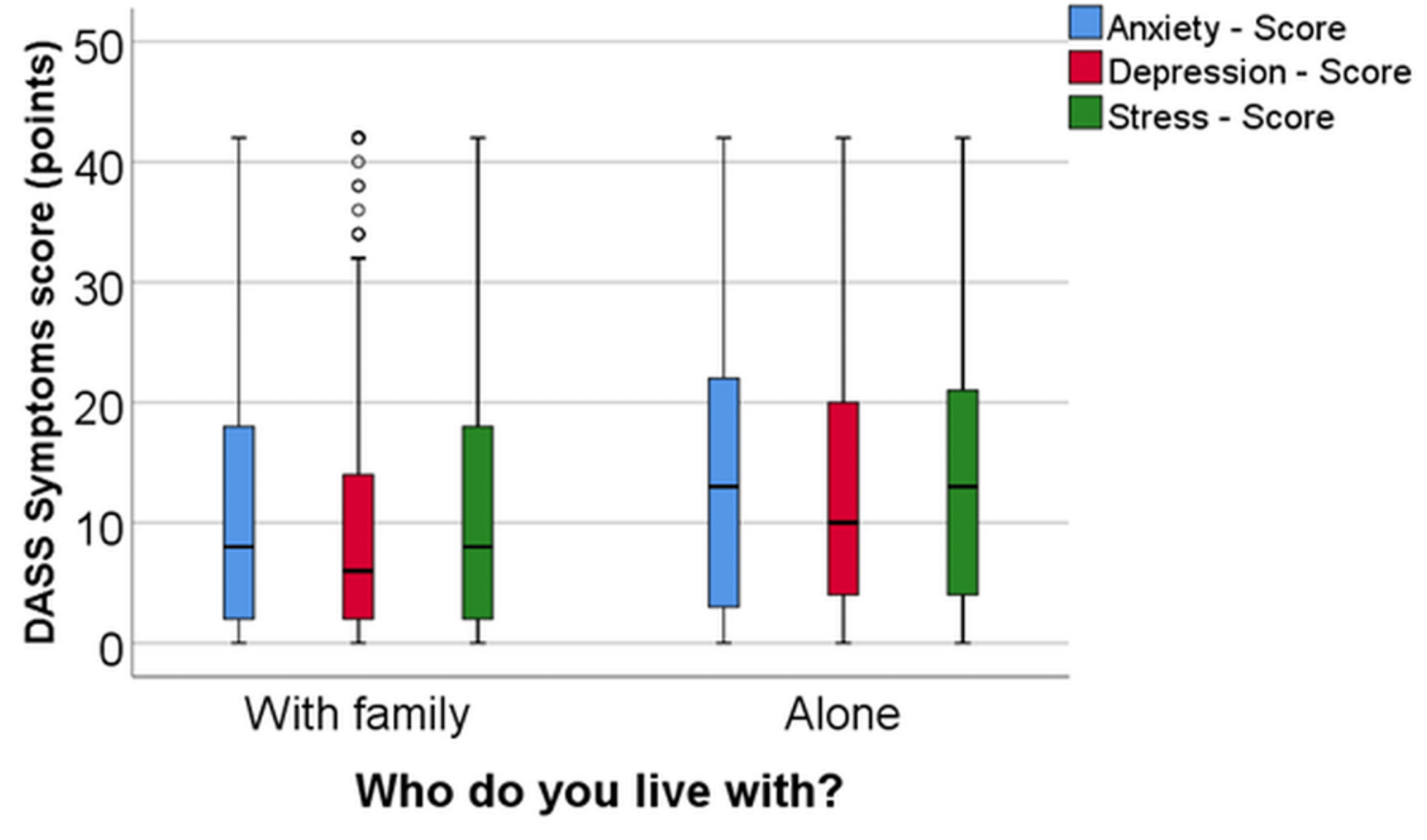
Analysis of anxiety, depression, and stress scores across different living arrangements DASS, Depression, Anxiety, and Stress Scale.

While 80.04% (n = 417) of respondents reside with at least one other person, 19.96% (n = 104) live alone. According to the Mann-Whitney U-tests, there were significant differences in the values for anxiety (p = 0.042), depression (p = 0.017), and stress (p = 0.021) between the groups. Patients living alone exhibited significantly higher values for the analyzed scores (anxiety: median = 13, IQR = 2.5-22; depression: median = 10, IQR = 4-20; stress: median = 13, IQR = 4-21.5) compared to patients who lived with their family (anxiety: median = 8, IQR = 2-18; depression: median = 6, IQR = 2-14; stress: median = 8, IQR = 2-18) (Figure 2).

When analyzing the impact of pets on the perception of DASS parameters, we observed an improvement in anxiety among pet owners, although without statistically significant relevance. The distribution of scores was non-parametric across all groups, as indicated by the Shapiro-Wilk tests (p < 0.05). According to the Mann-Whitney U tests, the differences in anxiety (p = 0.575), depression (p = 0.901), and stress (p = 0.733) scores were not statistically significant between persons with and without pets.

Comparative analysis of anxiety, depression, and stress levels among groups that had experienced the loss of a relative or a friend

The loss through the death of a person with whom there were channels of affective communication and a history of moments filled with intense positive emotion will result in the suffering of the social circle and mourning. The study aimed to identify the extent of these sufferings in the context of the COVID-19 pandemic. Based on initial results, 6.14% (n = 32) of respondents reported having first-degree relatives who had died from this disease.

According to the Mann-Whitney U-tests, there were significant differences in the values for anxiety (p < 0.001), depression (p < 0.001), and stress (p < 0.001) between the groups. Patients who had lost first-degree relatives exhibited significantly higher values for the analyzed scores (anxiety: median = 22, IQR = 10.5-32; depression: median = 19, IQR = 10.5-30; stress: median = 22, IQR = 9-33.5) compared to patients who had not lost relatives (anxiety: median = 8, IQR = 2-18; depression: median = 6, IQR = 2-16; stress: median = 8, IQR = 2-18).

Additionally, the death of a friend due to COVID-19 has destabilized the mental equilibrium of respondents compared to individuals who have not experienced grief. In our study, 33.59% (n = 175) of respondents reported losing a friend.

According to the Mann-Whitney U-tests, there were significant differences in the values for anxiety (p < 0.001), depression (p < 0.001), and stress (p < 0.001) between the groups. Patients who had lost friends exhibited significantly higher values for the analyzed scores (anxiety: median = 14, IQR = 4-26; depression: median = 12, IQR = 2-22; stress: median = 14, IQR = 4-24) compared to patients who had not lost close individuals (anxiety: median = 8, IQR = 2-16.5; depression: median = 6, IQR = 2-14; stress: median = 8, IQR = 2-16).

Comparison of anxiety, depression, and stress scores between the COVID-19 vaccination groups

The advent of the vaccine has positively altered the overall mental state, instilling hope for halting the spread of the virus and associated pathologies. The analysis of DASS variables aimed to quantify these psychological changes for the analyzed population. The distribution of scores was non-parametric across all groups, as indicated by the Shapiro-Wilk tests (p < 0.05).

Of the respondents, 75.43% (n = 393) were vaccinated, while 24.56% (n = 128) did not get the vaccine. Among the vaccinated, 27.25% (n = 142) received the vaccine before contracting COVID-19, and 9.78% (n = 51) were vaccinated after the COVID-19 infection. According to the Mann-Whitney U-tests, differences in anxiety (p = 0.211), depression (p = 0.158), and stress (p = 0.218) scores were not statistically significant between patients who were or were not vaccinated against SARS-CoV-2.

Comparative analysis of anxiety, depression, and stress levels between COVID-19 hospitalized patients and those managed outside hospital settings

**Figure 3:**
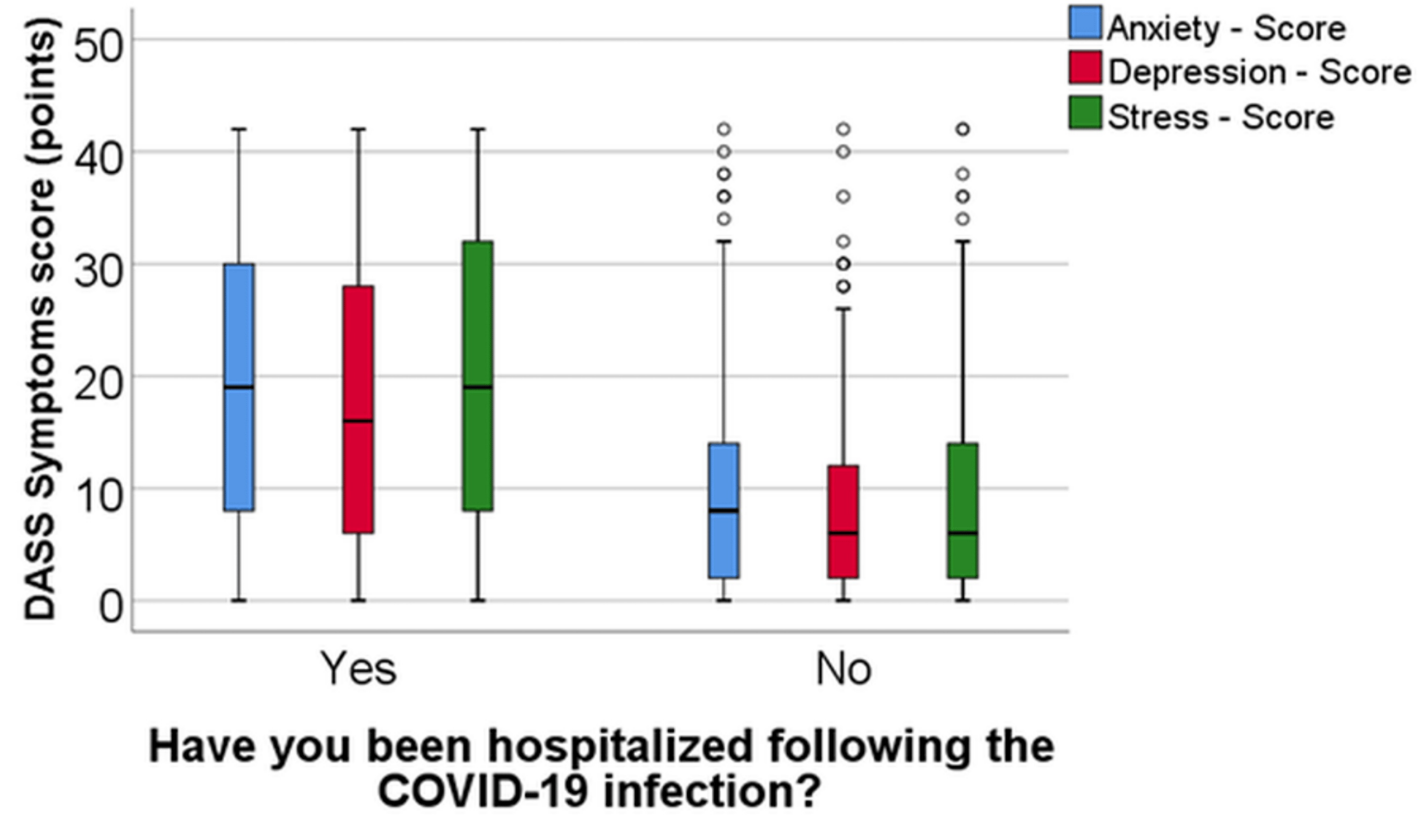
Comparison of anxiety, depression, and stress scores between the hospitalized groups DASS, Depression, Anxiety, and Stress Scale.

In the initial phase of the pandemic, patients were hospitalized for better isolation and to limit the spread of the virus in Romania. Subsequently, as the number of infection cases increased, COVID-19-positive patients were hospitalized for severe forms of the disease, requiring increased oxygen or even mechanical ventilation, necessitating invasive treatments. Moreover, individuals with chronic pathologies and compromised immune systems developed more severe forms of the illness. In this context, the daily governmental mortality reports were related to hospitalized patients. Against this factual backdrop, our study aimed to retrospectively assess the perception of COVID-19 patients regarding their hospitalization (Table 2, Figure 3).

**Table 2:** Comparison of anxiety, depression, and stress levels between the hospitalized groups *Mann-Whitney U-test, **Shapiro-Wilk test.

Hospitalization anxiety score	Average ± SD	Median (IQR)	Average rank	p-Value*
Present (p < 0.001**)	19.75 ± 13.22	19 (8-30)	345.43	<0.001
Absent (p < 0.001**)	9.51 ± 9.18	8 (2-14)	226.07	
Hospitalization/Depression score	Average ± SD	Median (IQR)	Average rank	p-Value*
Present (p < 0.001**)	18.01 ± 12.94	16 (6-28)	351.07	<0.001
Absent (p < 0.001**)	7.55 ± 7.83	6 (2-12)	223.78	
Hospitalization/Stress score	Average ± SD	Median (IQR)	Average rank	p-Value*
Present (p < 0.001**)	19.85 ± 13.55	19 (8-32)	348.89	<0.001
Absent (p < 0.001**)	8.93 ± 8.76	6 (2-14)	224.67	

The scores exhibited a non-parametric distribution across all groups, as determined by the Shapiro-Wilk tests (p < 0.05). According to the Mann-Whitney U-tests, significant differences were observed in anxiety (p < 0.001), depression (p < 0.001), and stress (p < 0.001) scores between the groups. Specifically, patients who were hospitalized following COVID-19 infection displayed markedly higher values for the analyzed scores (anxiety: median = 19, IQR = 8-30; depression: median = 16, IQR = 6-28; stress: median = 19, IQR = 8-32) compared to patients who were not hospitalized (anxiety: median = 8, IQR = 2-14; depression: median = 6, IQR = 2-12; stress: median = 6, IQR = 2-14).

Comparative analysis of anxiety, depression, and stress levels between the time of infection

1. The Initial Phase: This pertains to the period from the onset of the first case of COVID-19 until January 2021.

2. The Middle Phase: This covers the timeframe from January 2021 to January 2022, aligning with the emergence of the Omicron variant.

3. The Final Phase: This phase spans from January 2022 until May 5, 2023, coinciding with the World Health Organization's (WHO) declaration that the pandemic no longer constitutes an international concern.

When comparing anxiety, depression, and stress scores between individuals with a COVID-19 infection at the beginning of the pandemic and those without, the results are as follows: The distribution of scores was non-parametric across all groups based on the Shapiro-Wilk tests (p < 0.05). According to the Mann-Whitney U-tests, there are significant differences in anxiety (p < 0.001), depression (p < 0.001), and stress (p < 0.001) scores between the groups. Patients who had a COVID-19 infection at the beginning of the pandemic had significantly higher values for the analyzed scores (anxiety: median = 20, IQR = 4-32; depression: median = 16, IQR = 6-29; stress: median = 20, IQR = 6-32) compared to patients who weren't infected at the beginning of the pandemic (anxiety: median = 10, IQR = 4-18; depression: median = 8, IQR = 4-16; stress: median = 10, IQR = 4-18).

In the middle period of the pandemic, the distribution of scores was non-parametric across all groups based on the Shapiro-Wilk tests (p < 0.05). According to the Mann-Whitney U-tests, differences in anxiety (p = 0.095) and depression (p = 0.065) were not statistically significant between patients who were or were not COVID-19 infected during this period. There were only tendencies toward statistical significance, indicating higher values of scores in cases of infected patients over non-infected patients. However, the stress score (p = 0.045) was significant between groups. Patients who had a COVID-19 infection in the middle period of the pandemic had significantly higher values of the stress score (median = 14, IQR = 5-22) compared to patients who weren't infected during this period (median = 10, IQR = 4-22). These results suggest that the impact of COVID-19 on the mental health of infected patients tends to decrease as time progresses from the beginning of the pandemic.

To analyze the ending phase of the pandemic, the distribution of scores displayed non-parametric characteristics across all groups, as indicated by the Shapiro-Wilk tests (p < 0.05). According to the Mann-Whitney U-tests, significant differences in anxiety (p = 0.042), depression (p = 0.007), and stress (p < 0.001) scores were observed between groups. Patients who contracted COVID-19 at the end of the pandemic exhibited notably lower values for the analyzed scores (anxiety: median = 10, IQR = 4-20; depression: median = 8, IQR = 4-14; stress: median = 8, IQR = 4-18) compared to patients who remained uninfected at the end of the pandemic (and were infected at the beginning and/or in the middle period) (anxiety: median = 14, IQR = 6-24; depression: median = 12, IQR = 4-22; stress: median = 14, IQR = 6-24). These results provide evidence that the mental health impact of COVID-19 infection diminishes even further over time, possibly reaching its lowest point at the end of the pandemic, primarily due to societal adaptation, milder forms of the disease, and effective treatments.

Comparative analysis of anxiety, depression, and stress levels concerning reinfection with SARS-CoV-2

In Table 3, the scores exhibited a non-parametric distribution across all groups as per the Shapiro-Wilk tests (p < 0.05). A total of 18.6% (n = 97) reported experiencing the disease on multiple occasions. Based on the Mann-Whitney U-tests, significant differences in anxiety (p = 0.001), depression (p < 0.001), and stress (p < 0.001) scores were observed between groups. Patients with reinfections had notably higher scores (anxiety: median = 14, IQR = 4-26; depression: median = 12, IQR = 4-24; stress: median = 14, IQR = 5-24) compared to those without reinfections (anxiety: median = 8, IQR = 2-18; depression: median = 6, IQR = 2-14; stress: median = 8, IQR = 2-18).

**Table 3:** Comparison of anxiety, depression, and stress scores between groups with and without reinfection *Mann-Whitney U-test, **Shapiro-Wilk test.

Reinfection/Anxiety score	Average ± SD	Median (IQR)	Average rank	p-Value*
Present (p < 0.001**)	16.39 ± 12.75	14 (4-26)	308.51	0.001
Absent (p < 0.001**)	11.57 ± 10.97	8 (2-18)	250.13	
Reinfection/Depression score	Average ± SD	Median (IQR)	Average rank	p-Value*
Present (p < 0.001**)	15.46 ± 12.07	12 (4-24)	329.52	<0.001
Absent (p < 0.001**)	9.46 ± 10.02	6 (2-14)	245.32	
Reinfection/Stress score	Average ± SD	Median (IQR)	Average rank	p-Value*
Present (p < 0.001**)	16.31 ± 12.75	14 (5-24)	314.79	<0.001
Absent (p < 0.001**)	11.12 ± 10.95	8 (2-18)	248.69	

Regression models

To consider employing a linear regression model for each of the analyzed scores, we summarize the significant associations demonstrated in the study between the scores and various factors.

The generalized linear models for predicting anxiety, depression, and stress are as follows.

The data from Tables 4-7 reveal the following results based on the multivariable generalized linear models:

· Patients at risk of higher anxiety scores included those from household backgrounds compared to medical staff (p = 0.037) or patients with other occupations (p = 0.030), those who experienced the loss of first-degree relatives (p < 0.001), and those who were hospitalized (p < 0.001).

· Patients at risk of higher depression scores were those from household backgrounds compared to medical staff (p = 0.041) or patients with other occupations (p = 0.033), individuals living alone (p = 0.021), those who experienced the loss of first-degree relatives (p < 0.001), loss of friends (p = 0.041), hospitalization (p = 0.040), and reinfection (p = 0.040).

· Patients at risk of higher stress scores were individuals living alone (p = 0.049), those who experienced the loss of first-degree relatives (p < 0.001), loss of friends (p = 0.037), hospitalization (p < 0.001), and those who were not infected with COVID-19 at the end of the pandemic but rather at the beginning or middle of it (p = 0.022).

**Table 4:** Summary of all significant factors associated with analyzed scores

Factor/Score association (p)	Anxiety	Depression	Stress
Occupation	0.040	0.049	0.027
Living arrangement	0.042	0.017	0.021
Loss - first degree	<0.001	<0.001	<0.001
Loss - friends	<0.001	<0.001	<0.001
Hospitalization	<0.001	<0.001	<0.001
COVID infection - pandemic end	0.042	0.007	<0.001
Reinfection	0.001	<0.001	<0.001

**Table 5:** Generalized linear models used in predicting anxiety score

Parameter	Univariable		Multivariable	
	B (95% CI)	p-Value	B (95% CI)	p-Value
Occupation				
Medical staff	-3.698 (-7.132 to -0.265)	0.035	-4.128 (-0.255 to -8.001)	0.037
Other	-4.736 (-7.794 to -1.678)	0.002	-3.937 (-0.392 to -7.482)	0.030
Household (reference)	-	-	-	-
Living with family	-2.755 (-5.204 to -0.305)	0.028	-2.586 (-5.38 to 0.207)	0.070
Loss - first-degree relatives	10.688 (6.696 to 14.681)	<0.001	8.754 (4.521 to 12.987)	<0.001
Loss - friends	5.197 (3.163 to 7.232)	<0.001	2.010 (-0.485 to 4.505)	0.114
Hospitalization	10.233 (8.244 to 12.223)	<0.001	8.198 (5.829 to 10.567)	<0.001
COVID infection - end	-2.976 (-5.47 to -0.482)	0.019	-1.512 (-3.773 to 0.749)	0.190
Reinfection	4.821 (2.328 to 7.314)	<0.001	1.400 (-1.132 to 3.933)	0.278

**Table 6:** Generalized linear models used in predicting depression score

Parameter	Univariable		Multivariable	
	B (95% CI)	p-Value	B (95% CI)	p-Value
Occupation				
Medical staff	-2.571 (-5.776 to 0.634)	0.116	-3.739 (-7.319 to -0.159)	0.041
Other	-3.850 (-6.704 to -0.995)	0.008	-3.573 (-6.850 to -0.296)	0.033
Household (reference)	-	-	-	-
Living with family	-3.293 (-5.568 to -1.018)	0.005	-3.050 (-5.633 to -0.468)	0.021
Loss - first-degree relatives	10.507 (6.798 to 14.216)	<0.001	8.196 (4.283 to 12.109)	<0.001
Loss - friends	5.053 (3.162 to 6.944)	<0.001	2.407 (0.101 to 4.714)	0.041
Hospitalization	10.467 (8.652 to 12.283)	<0.001	8.069 (5.879 to 10.258)	<0.001
COVID infection - end	-3.261 (-5.619 to -0.902)	0.007	-1.809 (-3.899 to 0.282)	0.090

**Table 7:** Generalized linear models used in predicting stress score

Parameter	Univariable		Multivariable	
	B (95% CI)	p-Value	B (95% CI)	p-Value
Occupation				
Medical staff	-2.524 (-5.965 to 0.918)	0.151	-2.834 (-6.695 to 1.027)	0.150
Other	-4.190 (-7.255 to -1.125)	0.007	-3.205 (-6.740 to 0.329)	0.076
Household (reference)	-	-	-	-
Living with family	-3.090 (-5.539 to -0.641)	0.013	-2.788 (-5.573 to -0.002)	0.049
Loss - first-degree relatives	10.098 (6.090 to 14.106)	<0.001	7.923 (3.703 to 12.143)	<0.001
Loss - friends	5.483 (3.452 to 7.514)	<0.001	2.651 (0.163 to 5.138)	0.037
Hospitalization	10.924 (8.959 to 12.888)	<0.001	8.569 (6.207 to 10.931)	<0.001
COVID infection - end	-4.247 (-6.747 to -1.748)	0.001	-2.634 (-4.889 to -0.380)	0.022
Reinfection	5.191 (2.702 to 7.681)	<0.001	1.356 (-1.169 to 3.881)	0.293

The key determinants contributing to increased scores across all mental health disorders, as indicated by Tables 4-7, were the loss of a first-degree relative and hospitalization due to COVID-19 pathology.

## Discussion

Recently, the onset of the COVID-19 pandemic has underscored the significant impact that a new virus can exert on human life. The substantial changes triggered by COVID-19 have presented notable societal challenges worldwide, influencing various facets of existence [1]. The protracted duration and far-reaching repercussions of this crisis have led to widespread psychological distress on a global scale.

Since the beginning of the pandemic, researchers have documented a decline in the general population's well-being, attributed to both the fear of illness and the secondary consequences of the pandemic, including imposed restrictions, and pre-existing economic, social, and medical impacts [19,20]. The present study aims to assess the mental state of the Romanian population post-pandemic, employing a validated psychological tool. Consequently, we intend to conduct a comparative analysis of the results obtained from the sampled population of similar literature.

Mental distress and occupational status

Many studies conducted across continents and throughout different phases of the pandemic have consistently shown, regardless of sociocultural context, that healthcare workers have been more profoundly affected in terms of psychological burden compared to individuals in other life domains [13,21,22]. Our study aligns with these global scientific findings. However, the novelty lies in the fact that, despite being exposed, medical professionals exhibited lower levels of anxiety, depression, and stress compared to those who were not working at all, including retirees, homemakers, or the unemployed. This may initially seem counterintuitive, but upon closer examination, it appears that from an external perspective, the battle seems more perilous than when you are a "soldier in the fight." One possible explanation is that active involvement in a concrete medical activity eliminates the fog of misinformation and conspiracy theories. Instead, the satisfaction of aiding those in distress emerges aspects that, overall, reduce the negative emotional burden compared to those who experienced the pandemic more through media reports, amplified by personal vulnerability, such as comorbidities increasing the risk of a more severe form of the disease, and/or financial and social insecurity.

When comparing the psychological strain results of workers in various non-medical fields with those of individuals who are not employed, our findings indicate that the active population expresses lower levels of depression, anxiety, and stress. Similar results, suggesting the activation of mental protective mechanisms for the employed population across different social layers, have been recently published [22,23]. These results emphasize that active engagement in a work regimen represents a resilience strategy and reduces the negative impact of uncertainty, stress, anxiety, and depression in the general population.

Living arrangement and its impact on pandemic time mental health

From the perspective of the Romanian individuals who were interviewed, living alone exacerbated stress, anxiety, and depression, regardless of other contributing factors. Similar effects have been reported by other researchers. For example, a Spanish study on medical professionals during the pandemic revealed that living alone does not significantly impact mental well-being; instead, it is primarily the experienced loneliness that has unfavorable effects [24].

In a study conducted in the United States, it was found that residing in larger families had a resilience effect during the pandemic, while living alone had a negative impact on mental well-being [25]. Additionally, in China, when factors such as age, gender, and social status were analyzed alongside living alone, female loneliness and self-employment were identified as variables that negatively influence psychosocial well-being [26].

In conclusion, living alone during the pandemic period had adverse effects on mental balance, irrespective of the continent or the population studied, and this aspect was further influenced by loneliness, as well as economic and medical conditions.

Some authors have demonstrated that the human-pet relationship acts as an emotional protective factor, even during the pandemic [27]. Analyzing this hypothesis, the current study was unable to establish statistically significant correlations between the presence of a pet and improvement in mental health status within the studied population.

The impact of grief on mental health

The death of a friend or a family member abruptly alters the psychological framework of life, as the grief caused by the irreversible loss of a person influences their attitude and behavior, and can also have significant somatic manifestations. In a large-scale Australian cohort, a longitudinal study spanning 15 years has determined that the degree of connection or attachment developed over time significantly influences the manifestation of anxiety symptoms following the loss of a friend. The effect of losing a family member is further shaped by additional variables such as age, gender, ethnicity, religion, and interpersonal dynamics. These elements can lead to diverse degrees of psychological distress across different types of loss. The impact may persist for up to four years, with females being more adversely affected than males [28]. To all these aspects, the anxiety from the pandemic, fear of illness, and social restrictions add further strain on human resilience [29,30].

A study encompassing 10 Latin American countries, where mortality rates during the initial wave of the pandemic reached alarming levels, investigated the effect of grief on health science students, quantifying the extent of distress about the emotional proximity to the deceased. The findings align with intuitive expectations: the closer the deceased was to the individuals, the deeper the impact on their stress, anxiety, and depression levels [31].

Our results are consistent with existing literature: individuals who have suffered the loss of a friend or a close family member display higher levels of anxiety, stress, and depression in comparison to those who have not experienced the death of a loved one. Thus, negative emotions associated with grief further destabilize individuals with a more fragile mental state, those with lower resilience to stress, or those with pre-existing medical conditions.

The influence of vaccination on mental health

The vaccine against SARS-CoV-2 was introduced with the hope of minimizing the somatic progression of the disease and, indirectly, optimizing the emotional mechanisms that govern the population's psychological state. However, the introduction of the vaccine triggered unexpected counter-reactions, ranging from hesitation and fear to outright refusal of vaccination in certain cases. The causes of this anxiety were linked to misinformation, the fact that the research behind the vaccine was not entirely transparent and that it was not extensively tested due to global emergency reasons, as we know [32]. In this context, studies have yielded inconsistent results regarding adherence to vaccination and the alleviation of anxiety. For example, in the US, vaccination improved psychological parameters such as anxiety, stress, and depression, except among certain ethnic groups and individuals with lower educational levels [33]. On the other hand, a study in China showed that the level of anxiety and depression was not alleviated by the number of vaccinations or the timing of vaccination [31]. A study conducted in Poland, which analyzed the attitudes of healthcare professionals toward vaccination, found that 94% of healthcare workers were open to the idea of vaccination, being informed and aware of the benefits provided. The authors examined the factors contributing to vaccine hesitancy, which was more prevalent among the general population, and identified misinformation, distrust in state institutions, and a lower level of education as key determinants [34].

In a 2021 UK study, researchers found significant mental health improvements following vaccination. These benefits were not only statistically significant but also substantial, akin to the effects of major life events. This indicates that before vaccination, widespread anxiety about catching COVID-19 was a major source of psychological distress for many. Additionally, the study emphasized that individuals with underlying health conditions (clinically vulnerable) and older adults particularly experienced significant mental health improvements from vaccination [27].

When we analyzed the Romanian group from the perspective of vaccination, although there were improvements in mental status, these did not yield statistically significant changes, regardless of whether the vaccine was received before or after experiencing COVID-19. These findings confirm the multifaceted nature of the psycho-emotional dynamics during the pandemic.

Comparative analysis of distress in the population requiring hospitalization versus those who did not

The findings of our study highlight the perception of stress, anxiety, and depression in COVID patients who required hospitalization in a statistically significant manner, compared to those who managed the illness at home. This aspect aligns with other published medical reports, regardless of the pandemic phase or nationality [35,36].

Starting from the host immune response to SARS-CoV-2 infection, the persistent psychological stress before and during infection, and a possible direct viral infection of the central nervous system, a group of Italian researchers have proposed the hypothesis of potential mechanisms inducing neuropsychiatric sequelae [37]. This concept is bolstered by findings that elevated immune/inflammatory baselines, marked by increased circulating inflammation biomarkers, are identified in mood disorders without apparent triggering events. These findings are currently explored as fundamental pathogenic mechanisms behind depressive disorders [38]. Peripheral cytokines involved in the host's anti-viral response may induce psychiatric symptoms by triggering inflammation both peripherally and in the central nervous system [37]. Moreover, major stress factors including the fear of severe and unfamiliar illnesses, isolation, and social stigma may play an important role in the extensive emotional turmoil and the heightened likelihood of mental health disorders among COVID-19 hospitalized patients.

Mental strain according to the timing of infection, concerning the pandemic waves

In our study, we found that patients who contracted COVID-19 at the beginning of the pandemic had higher levels of anxiety, depression, and stress. These psychological symptoms gradually decreased as the pandemic progressed. The mitigation of the psychological impact on the population has several causes: first, the emergence of effective therapeutic strategies; second, the decrease in the virulence of the pathogen over time; and third, the development of adaptation mechanisms and mental flexibility to the imposed regulations.

In Italy, research focused on psychological distress during the first wave of the COVID-19 lockdown assessed levels of stress, depression, and anxiety at the beginning and end of the lockdown period, spanning two months. The study revealed that anxiety levels remained relatively unchanged, whereas stress and depression levels increased over this period. Additionally, individuals with initially higher depression and stress levels were found to be more likely to report elevated levels at the end of the lockdown [39].

Contrary to the results from Italy, a longitudinal study of two UK cohorts indicates that depression rates during the pandemic mirrored those before the pandemic. However, the rate of anxiety almost doubled, compared to the pre-pandemic times. For both cohorts, the levels of anxiety and depression during the pandemic were elevated in younger people, women, individuals with existing mental or physical health issues, and those experiencing socio-economic challenges [5].

To reduce the contradiction, the variable of time must be introduced, in the sense that the Italian study was published in November 2020, and the British study in June 2021, both pieces of research being conducted during the pandemic when the psychological impact was fully felt by the population.

A longitudinal study on the mental health effects of the COVID-19 pandemic among middle-aged and older Canadian adults reveals that compared to before the pandemic, adults had double the likelihood of experiencing depressive symptoms. Those with lower socioeconomic status and poorer health were particularly affected. Initially, over 43% of adults showed moderate to high levels of depressive symptoms, which increased over time. Loneliness and pandemic-related stressors predicted worsening depressive symptoms [40].

The consequences of reinfection

Reinfection with COVID-19, defined as a positive RT-PCR test occurring at least 90 days after the primary infection, was initially rare; the first documented case was reported in Hong Kong, China, in August 2020, after 142 days had passed between infections [41].

A meta-analysis on COVID-19 reinfection, published in 2022 and analyzing 91 studies, concludes that strong natural immunity develops after the primary infection and can persist for more than a year. Even though reinfection rates notably increased during the Omicron variant surge, the probability of encountering a severe or deadly disease from a subsequent infection stayed exceedingly low [42].

Another meta-analysis published in 2023 in a Chinese journal concludes that, although the possibility of reinfection exists, it affects the general population at a rate of less than 3%, and healthcare workers at a rate of 6.02% [43].

A study from the US, utilizing the Department of Veterans Affairs national healthcare database, found that reinfection with SARS-CoV-2 poses additional risks of mortality, hospitalization, and various sequelae, including issues related to the lungs, heart, blood, diabetes, gastrointestinal system, kidneys, mental health, muscles and bones, and nervous system. These risks were observed regardless of one's vaccination status and were most significant during the acute phase but continued to be present six months into the post-acute phase. Compared to individuals who were never infected, the cumulative risks and health impacts escalated with each subsequent infection. Therefore, strategies to prevent reinfection are essential to reduce the overall disease burden caused by SARS-CoV-2 [11].

The results of a systematic review on SARS-CoV-2 reinfection indicate that reinfection is possible either due to the host immune system's inability or the aggressiveness of other viral variants. However, there is also published literature that identifies reinfection solely based on RT-PCR without viral genetic sequencing, which introduces a source of bias in validating conclusions. Consequently, the authors recommend that patients who have recovered from the disease and have been vaccinated continue to adhere to biological safety measures [44].

The psychological effects of reinfection, especially in Romania, have not yet been sufficiently researched. In our study group, only 18.6% of participants reported having been infected with SARS-CoV-2 multiple times, and these individuals had higher levels of anxiety, depression, and stress compared to those who had not been repeatedly infected.

In summary, reinfection with COVID-19 poses risks of both physical and psychological disability, with the mental health component acting like a difficult-to-estimate halo in terms of magnitude, yet having a long-term influence on the well-being of the global population.

The limitations of the study and future directions

The present study, while contributing valuable insights, does have a few limitations that may provide opportunities for future research. First, the use of a cross-sectional design in this study does not enable the interpretation of the findings as implying causality. Longitudinal studies are needed to identify how mental health outcomes change over time and the causal factors of these changes. Second, this study was conducted on a convenience sample, which limits the generalizability of the findings. The participants were selected based on their use of the internet and technology, resulting in the underrepresentation of elderly and socio-economically disadvantaged populations. Further studies are warranted to examine mental health using more representative samples. The third sensitive point involves personal perception and the anonymous declaration of the absence of prior mental health conditions; the authors could not verify the accuracy of this information, relying instead on the honesty of the participants. Finally, self-assessment of COVID-19 infection/reinfection and its severity may introduce biases that could either understate or overstate symptoms. Self-report biases and poor recall should, therefore, be considered when interpreting the findings.

Continued investigation is crucial for delving into the interplay and impact of pre-existing and COVID-19-specific factors on mental health during the pandemic. Monitoring trends in depression and anxiety, along with their related disruptions, is key for evaluating the enduring effects of the crisis. Such initiatives are vital for crafting future approaches that effectively safeguard mental and physical health. Embracing longitudinal studies over several years will furnish a deeper insight into the mental health status across populations. Incorporating longitudinal designs along with a blend of qualitative and quantitative methodologies will address current limitations and enhance the understanding of the psychological impact stemming from the COVID-19 pandemic on the Romanian population. This comprehensive strategy aims to unravel the full scope of the psychological burden brought on by the pandemic, ensuring a well-rounded perspective on mental health trends and aiding in the development of targeted interventions.

## Conclusions

The pandemic has led to significant psychological distress, manifesting as anxiety, stress, and depression, often reaching levels of clinical concern. Therefore, addressing and reducing the negative psychological effects of COVID-19 should be an urgent public health priority. Understanding these psychological impacts is crucial for providing appropriate support and care to those at risk. In the socioeconomic context of Romania, we have identified that elderly individuals, retirees, or those unemployed, who live alone, have been hospitalized for COVID-19, have suffered the loss of a loved one during the pandemic, and have experienced reinfection, regardless of vaccination status, remain psychologically vulnerable even after the pandemic has ended.

## Appendices

**Table 8:** Questionnaire: The Mental Burden of the COVID-19 Pandemic: A Retrospective Post-Pandemic View From a Romanian Sample

GENERAL INFORMATION	
Gender	Male
	Female
Age	
Occupation	
With whom do you live?	Alone
	With parents
	With spouse
	With spouse and children
	With children
	Other option
Have you been vaccinated against COVID-19?	Yes
	No
Were you vaccinated prior to COVID-19 infection?	Yes
	No
	Pre- and post-infection
DASS-21 The following questions will be filled out by thinking about how you felt when you had the SARS-CoV-2 viral infection, when restriction measures were imposed, and when the COVID-19 pandemic started. Please read each statement and choose a corresponding number 0, 1, 2, or 3, indicating how much the situation applied to you. There are no right or wrong answers. Don't spend too much time on each statement. • Rate as follows: 0 - Did not apply to me at all – NEVER; 1 - Applied to me to some degree, or some of the time - SOMETIMES; 2 - Applied to me to a considerable degree, or a good part of time - OFTEN; 3 - Applied to me very much, or most of the time - ALMOST ALWAYS	
I found it hard to wind down	3 - Applied to me very much, or most of the time - ALMOST ALWAYS
	2 - Applied to me to a considerable degree, or a good part of time - OFTEN
	1 - Applied to me to some degree, or some of the time - SOMETIMES
	0 - Did not apply to me at all - NEVER
I was aware of dryness of my mouth	3 - Applied to me very much, or most of the time - ALMOST ALWAYS
	2 - Applied to me to a considerable degree, or a good part of time - OFTEN
	1 - Applied to me to some degree, or some of the time - SOMETIMES
	0 - Did not apply to me at all - NEVER
I couldn’t seem to experience any positive feeling at all	3 - Applied to me very much, or most of the time - ALMOST ALWAYS
	2 - Applied to me to a considerable degree, or a good part of time - OFTEN
	1 - Applied to me to some degree, or some of the time - SOMETIMES
	0 - Did not apply to me at all - NEVER
I experienced breathing difficulty (e.g., excessively rapid breathing, breathlessness in the absence of physical exertion)	3 - Applied to me very much, or most of the time - ALMOST ALWAYS
	2 - Applied to me to a considerable degree, or a good part of time - OFTEN
	1 - Applied to me to some degree, or some of the time - SOMETIMES
	0 - Did not apply to me at all - NEVER
I found it difficult to work up the initiative to do things	3 - Applied to me very much, or most of the time - ALMOST ALWAYS
	2 - Applied to me to a considerable degree, or a good part of time - OFTEN
	1 - Applied to me to some degree, or some of the time - SOMETIMES
	0 - Did not apply to me at all - NEVER
I tended to over-react to situations	3 - Applied to me very much, or most of the time - ALMOST ALWAYS
	2 - Applied to me to a considerable degree, or a good part of time - OFTEN
	1 - Applied to me to some degree, or some of the time - SOMETIMES
	0 - Did not apply to me at all - NEVER
I experienced trembling (e.g., in the hands)	3 - Applied to me very much, or most of the time - ALMOST ALWAYS
	2 - Applied to me to a considerable degree, or a good part of time - OFTEN
	1 - Applied to me to some degree, or some of the time - SOMETIMES
	0 - Did not apply to me at all - NEVER
I felt that I was using a lot of nervous energy	3 - Applied to me very much, or most of the time - ALMOST ALWAYS
	2 - Applied to me to a considerable degree, or a good part of time - OFTEN
	1 - Applied to me to some degree, or some of the time - SOMETIMES
	0 - Did not apply to me at all - NEVER
I was worried about situations in which I might panic and make a fool of myself	3 - Applied to me very much, or most of the time - ALMOST ALWAYS
	2 - Applied to me to a considerable degree, or a good part of time - OFTEN
	1 - Applied to me to some degree, or some of the time - SOMETIMES
	0 - Did not apply to me at all - NEVER
I felt that I had nothing to look forward to	3 - Applied to me very much, or most of the time - ALMOST ALWAYS
	2 - Applied to me to a considerable degree, or a good part of time - OFTEN
	1 - Applied to me to some degree, or some of the time - SOMETIMES
	0 - Did not apply to me at all - NEVER
I found myself getting agitated	3 - Applied to me very much, or most of the time - ALMOST ALWAYS
	2 - Applied to me to a considerable degree, or a good part of time - OFTEN
	1 - Applied to me to some degree, or some of the time - SOMETIMES
	0 - Did not apply to me at all - NEVER
I found it difficult to relax	3 - Applied to me very much, or most of the time - ALMOST ALWAYS
	2 - Applied to me to a considerable degree, or a good part of time - OFTEN
	1 - Applied to me to some degree, or some of the time - SOMETIMES
	0 - Did not apply to me at all - NEVER
I felt down-hearted and blue	3 - Applied to me very much, or most of the time - ALMOST ALWAYS
	2 - Applied to me to a considerable degree, or a good part of time - OFTEN
	1 - Applied to me to some degree, or some of the time - SOMETIMES
	0 - Did not apply to me at all - NEVER
I was intolerant of anything that kept me from getting on with what I was doing	3 - Applied to me very much, or most of the time - ALMOST ALWAYS
	2 - Applied to me to a considerable degree, or a good part of time - OFTEN
	1 - Applied to me to some degree, or some of the time - SOMETIMES
	0 - Did not apply to me at all - NEVER
I felt I was close to panic	3 - Applied to me very much, or most of the time - ALMOST ALWAYS
	2 - Applied to me to a considerable degree, or a good part of time - OFTEN
	1 - Applied to me to some degree, or some of the time - SOMETIMES
	0 - Did not apply to me at all - NEVER
I was unable to become enthusiastic about anything	3 - Applied to me very much, or most of the time - ALMOST ALWAYS
	2 - Applied to me to a considerable degree, or a good part of time - OFTEN
	1 - Applied to me to some degree, or some of the time - SOMETIMES
	0 - Did not apply to me at all - NEVER
I felt I wasn’t worth much as a person	3 - Applied to me very much, or most of the time - ALMOST ALWAYS
	2 - Applied to me to a considerable degree, or a good part of time - OFTEN
	1 - Applied to me to some degree, or some of the time - SOMETIMES
	0 - Did not apply to me at all - NEVER
I felt that I was rather touchy	3 - Applied to me very much, or most of the time - ALMOST ALWAYS
	2 - Applied to me to a considerable degree, or a good part of time - OFTEN
	1 - Applied to me to some degree, or some of the time - SOMETIMES
	0 - Did not apply to me at all - NEVER
I was aware of the action of my heart in the absence of physical exertion (e.g., sense of heart rate increase, heart missing a beat)	3 - Applied to me very much, or most of the time - ALMOST ALWAYS
	2 - Applied to me to a considerable degree, or a good part of time - OFTEN
	1 - Applied to me to some degree, or some of the time - SOMETIMES
	0 - Did not apply to me at all - NEVER
I felt scared without any good reason	3 - Applied to me very much, or most of the time - ALMOST ALWAYS
	2 - Applied to me to a considerable degree, or a good part of time - OFTEN
	1 - Applied to me to some degree, or some of the time - SOMETIMES
	0 - Did not apply to me at all - NEVER
I felt that life was meaningless	3 - Applied to me very much, or most of the time - ALMOST ALWAYS
	2 - Applied to me to a considerable degree, or a good part of time - OFTEN
	1 - Applied to me to some degree, or some of the time - SOMETIMES
	0 - Did not apply to me at all - NEVER
PANDEMIC/SYMPTOMS	
When did the infection occur?	At the beginning of the pandemic
	In the middle of the pandemic
	Near the end of the pandemic
Have you experienced the disease multiple times?/Have you had multiple reinfections?	Yes
	No
Were the second/other reinfections easier to tolerate?	Yes
	No
Did you have pre-existing comorbidities at the time of infection?	Yes
	No
If yes, what are they?	
Were you hospitalized due to COVID-19 infection?	Yes
	No
If yes, specify the form.	Mild
	Moderate
	Severe
Were you afraid of hospitalization?	Yes
	No
Have first-degree relatives died as a result of this disease?	Yes
	No
Have friends/close individuals died as a result of this disease?	Yes
	No
